# Single nucleotide polymorphisms in apoptosis pathway are associated with response to imatinib therapy in chronic myeloid leukemia

**DOI:** 10.1186/s12967-016-0837-5

**Published:** 2016-03-24

**Authors:** Qiaoli Zheng, Jiang Cao, Nada Hamad, Hyeoung-Joon Kim, Joon Ho Moon, Sang Kyun Sohn, Chul Won Jung, Jeffrey H. Lipton, Dennis Dong Hwan Kim

**Affiliations:** Department of Dermatology, Sir Run Run Shaw Hospital, School of Medicine, Zhejiang University, Hangzhou, Zhejiang Province China; Clinical Research Center, The Second Affiliated Hospital, School of Medicine, Zhejiang University, 88 Jiefang Road, Hangzhou, 310009 Zhejiang Province China; Department of Medical Oncology and Hematology, Princess Margaret Cancer Centre, University Health Network, University of Toronto, Toronto, Canada; Department of Medicine, University of Toronto, Toronto, Canada; Department of Hematology/Oncology, Chonnam National University Hwasun Hospital, Chonnam National University, Hwasun, South Korea; Department of Hematology/Oncology, Kyungpook National University Hospital, Kyungpook National University, Daegu, South Korea; Division of Hematology-Oncology, Department of Medicine, Samsung Medical Center, Sungkyunkwan University School of Medicine, Seoul, South Korea

**Keywords:** Single nucleotide polymorphisms, Imatinib, Chronic myeloid leukemia, Apoptosis pathway

## Abstract

**Background:**

The mechanism of action of imatinib is known to involve the Fas-mediated apoptosis pathway. Consequently inter-individual variations in this apoptosis pathway might be associated with imatinib response or resistance.

**Methods:**

This study attempted to focus on eight genotypes in the apoptosis pathway including *FAS* (rs1800682, rs2229521, rs2234767 and rs2234978), *FASLG* (rs763110), *CASP10* (rs13006529), and *APAF1* (rs1439123, rs2288713) and analyzed their association with treatment outcomes including molecular response with 4.5 log reduction (MR4.5), following imatinib therapy in 187 Korean CML patients.

**Results:**

The GG/GA genotype in *FAS* (rs2234767) showed a higher rate of MR4.5 than the AA genotype (at 5 years 59.7 vs 37.4 %, p = 0.013). Using a bootstrap procedure for internal validation we confirmed that FAS (rs2234767) correlates with MR4.5 (p = 0.050). Multivariate analysis confirmed that the *FAS* genotype (rs2234767) is an independent surrogate for MR4.5 (p = 0.019, HR 0.43, 95 % CI [0.22–0.87]).

**Conclusions:**

The Fas/FasL signaling pathway may represent the major pathway that mediates apoptosis in CML treated with imatinib. SNP markers in the apoptosis pathway including *FAS* genotype (rs2234767) can be potential surrogates for predicting deeper molecular response after imatinib therapy.

**Electronic supplementary material:**

The online version of this article (doi:10.1186/s12967-016-0837-5) contains supplementary material, which is available to authorized users.

## Background

Chronic myeloid leukemia (CML) is a clonal disorder characterized by the Philadelphia (Ph) chromosome as a result of translocation of chromosome 9 and 22, t(9; 22) (q34; q11) [1]. The molecular consequence of this translocation is rearrangement of the *BCR*-*ABL1* oncogene that encodes the chimeric bcr-abl1 protein with constitutive kinase activity [1]. This leads to increased proliferation and enhanced survival of leukemic stem cells (LSCs) [2]. The bcr-abl1 fusion protein enhances cell survival and exerts antiapoptotic activity in CML cells, thus mediating resistance to apoptosis [3–8]. Bcr-abl1 induces Bcl-XL, an antiapoptotic protein, through STAT5 phosphorylation [9]. The bcr-abl1 fusion protein also blocks the mitochondrial release of cytochrome C, exerting anti-apoptotic activity [10, 11] and inhibits other proapoptotic proteins including Bad or Bim [12–15].

Imatinib is a specific Abl-tyrosine kinase inhibitor that inhibits cellular growth and induces apoptosis in CML [16]. Once imatinib binds to the bcr-abl1 oncoprotein, it inactivates the kinase activity and subsequent signal transduction pathway resulting in apoptosis [17–19]. Imatinib can also restore Bad and Bim, which are inhibited by the bcr-abl1 fusion protein [12–15]. The primitive quiescent Philadelphia positive LSCs are relatively insensitive to imatinib or other tyrosine kinase inhibitors (TKIs) [20, 21], so it is extremely difficult to eradicate them with imatinib or other TKIs.

The Fas-mediated apoptosis pathway plays an important role in imatinib’s mechanism of action. Cells undergo apoptosis in response to signals through several different mechanisms including the Fas receptor (Fas-R) pathway. The Fas-R is expressed on hematopoietic stem cells (HSCs) in CML patients [22]. The Fas-induced pathway can trigger apoptotic signals to both normal HSCs and CML cells [22]. The Fas-R is upregulated by IFN-gamma and TNF-alpha on CD34+ cells and by IFN-alpha [23]. A recent study demonstrated that IFN-alpha treatment promotes proliferation of dormant HSCs, increasing the chance of G0 cells entering into the active cell division cycle [24]. IFN-alpha increases cell death in CML patients through the Fas-mediated apoptosis pathway by increasing Fas-R expression on LSCs and increasing their exposure to cytotoxic therapy including TKIs.

We postulated that inter-individual variation in the apoptosis pathway might be associated with imatinib response or resistance particularly in relation to the depth of molecular response (4.5 log reduction or MR4.5), which reflects LSC clearance by TKI therapy. We also attempted to identify predictive/prognostic genetic markers in CML patients treated with imatinib. In the current study, candidate genotypes were selected based on the literature. For SNP information was not available in the literature, SNPs were selected using the criteria of synonymous or non-synonymous SNP in exon region with minor allele frequency >1 %. We examined 8 apoptosis-associated SNPs and analyzed their association with response and resistance to imatinib in 187 Korean CML patients.

## Methods

### Study population

This study was performed according to the declaration of Helsinki. The study protocol was approved by the Institutional Research Board of the Sungkyunkwan University School of Medicine, Seoul, Korea. The study included 187 consecutive CML patients who started imatinib therapy between March 2002 and December 2008 in three centres in Korea with samples available for genotyping. Clinical information was obtained by retrospective medical chart review. Informed written consents were obtained from the participants in accordance with the requirements of the institutional Research Board of the Sungkyunkwan University School of Medicine. Biospecimens for genotyping were obtained from archived marrow or peripheral blood samples taken at the time of diagnosis.

### Patient evaluation and disease monitoring

Prior to imatinib therapy all patients had routine history taken, a physical examination, a complete blood count, standard baseline biochemistry tests and bone marrow evaluation for morphology, conventional cytogenetic analysis, and BCR/ABL mRNA RT-PCR. Cytogenetic analysis was performed using the G-banding technique. Patients were monitored regularly on an out-patient basis as follows: biweekly physical examinations, blood counts, and biochemistry were obtained during the first month of imatinib therapy, then monthly until a cytogenetic response was achieved, and then every 3 months thereafter. Bone marrow evaluation and/or FISH studies were performed every 3 months until a complete cytogenetic response was confirmed. Quantitative BCR/ABL mRNA PCR on peripheral blood was repeated every 3–4 months regardless of cytogenetic response. This was performed according to the manufacturer’s instructions using ABI 7900 Thermal Cycler (Applied Biosystems, Foster City, CA, USA). *ABL1* gene was used as a reference. The *BCR*-*ABL1* transcript level data was retrospectively compared and validated with those using Light Cycler. Standardization procedure to international scale was conducted per recommendation [25]. Sensitivity of the assay was up to 4.5 log reduction with minimum number of reference gene transcripts of 32,000 copies *ABL1*. Abl1 tyrosine kinase domain mutations were screened in any patient in an advanced phase of disease. Mutation screening was indicated in patients on imatinib who had evidence of treatment failure, loss of response or disease progression.

### Sequenom massARRAY genotyping system

Candidate genotypes were selected as synonymous or non-synonymous SNPs in exon regions with a minor allele frequency over 0.01 or based on the literature review. If the frequency was not available, it was sourced from the Entrez SNP site (http://www.ncbi.nlm.nih.gov/sites/entrez). The study included eight SNPs in the apoptosis pathway (Table 1), and their linkage disequilibrium plot is illustrated in Additional file 1: Figure S1.

**Table 1:** Summary of the candidate gene single nucleotide polymorphisms involved in the apoptosis pathway

Gene	Gene description	Chromosome	SNP ID	Allele (m/M)	Call rate (%)	MAF	HWE p value
*APAF1*	Apoptotic peptidase activating factor 1	12	rs1439123	C/T (Intron)	100	0.04	1.00
		12	rs2288713	G/T (Intron)	100	0.27	0.71
*CASP10*	Caspase 10, apoptosis-related cysteine peptidase	2	rs13006529	A/T (Leu → Ile)	100	0.18	1.00
*FASLG*	Fas ligand (TNF receptor superfamily member 6)	1	rs763110	T/C	98.4	0.29	0.98
*FAS*	Fas cell surface death receptor	10	rs1800682	T/C (Intron)	93.6	0.48	0.002
		10	rs2229521	G/A(Thr → Thr)	100	0.01	1.00
		10	rs2234767	A/G (Ala → Thr)	100	0.47	0.02
		10	rs2234978	T/C (Thr → Thr)	99.5	0.02	1.00

*m* minor allele, *M* major allele, *MAF* minor allele frequency, *HWE* Hardy–Weinberg equilibrium

First, genotyping was undertaken using the Sequenom^®^ iPLEX platform™, according to the manufacturer’s instructions (http://www.sequenom.com; Sequenom Inc, San Diego, CA, USA). DNA was extracted using the QIAGEN DNA purification Kit (Gentra Systems Inc, Minneapolis, MN, USA). We detected SNPs by analysis of primer extension products generated from previously amplified genomic DNA using a Sequenom chip-based matrix-assisted laser desorption/ionization time-of-flight (MALDI-TOF) mass spectrometry platform. Multiplex SNP assays were designed using SpectroDesigner software (Sequenom). Ninety-six well plates containing 2.5 ng DNA in each well were amplified by PCR following the specifications of Sequenom. Unincorporated nucleotides in the PCR product were deactivated using shrimp alkaline phosphatase. Allele discrimination reactions were performed by adding extension primer(s), DNA polymerase, and a cocktail mixture of deoxynucleotide triphosphates and di-deoxynucleotide triphosphates to each well. Mass Extend clean resin (Sequenom) was added to the mixture for removal of extraneous salts that might interfere with MALDI-TOF analysis. Primer extension products were then cleaned and spotted onto a SpectroChip. Genotypes were identified by spotting an aliquot of each sample onto a 96 SpectroChip (Sequenom), which was subsequently read by a MALDI-TOF mass spectrometer. Duplicate samples and negative controls were included for evaluation of genotyping quality. Primer sequences are listed in Additional file 1: Table S1. Genotyping was performed at Bioneer inc., Chung won, Korea.

### Definition of response criteria and end points

Previously defined response criteria to imatinib were used [26–28]. The response was determined retrospectively through chart review. A hematologic response was defined as normalized peripheral blood cell counts (WBC <10 × 10^9^/L and platelet <450 × 10^9^/L) without evidence of peripheral blasts, promyelocytes, or myelocytes, and without evidence of extramedullary disease including disappearance of palpable splenomegaly lasting for at least 4 weeks. Cytogenetic responses were categorized as complete (CCR; 0 % Ph^+^ cells in marrow by conventional cytogenetics or FISH), partial (1–34 % Ph^+^ cells in marrow), or minor (35–65 % Ph^+^ cells in marrow). A major cytogenetic response (MCR) was defined as the sum of CCR and partial cytogenetic response (0–34 % Ph^+^ cells in marrow). A major molecular response (MMR) was defined as less than 0.1 % of the *BCR/ABL* fusion gene transcript level on an international scale by quantitative PCR, and molecular response with 4.5 log reduction (MR4.5) was defined as disappearance of detectable *BCR/ABL* fusion gene transcripts, equivalent to less than 0.0032 % on the international scale.

Time to treatment failure (TF) was defined as the interval between initiation of imatinib therapy and occurrence of imatinib failure, including primary and secondary resistance, (i.e. loss of response (LOR). Time of LOR was defined as the interval between the date of any confirmed response (i.e. at least partial CR or deeper response) and the date at which criteria for response were no longer being met, including: transformation from chronic phase (CP) to accelerated phase (AP) or blastic crisis (BC), loss of CCR/MCR, and development of the Abelson tyrosine kinase domain mutation. Time to progression free survival (PFS) was defined as the interval between initiation of imatinib therapy and confirmation of progression to AP or BC, or death from any cause, while overall survival (OS) was defined as time from initiation of imatinib therapy until time of death from any cause or time of last follow-up. Primary resistance includes primary hematologic or cytogenetic response while secondary resistance includes loss of MCR/CCR, development of progression to advanced disease as well as development of TKD mutation.

### Statistical analysis

The candidate SNPs were primarily evaluated for adequacy of Hardy–Weinberg Equilibrium (HWE) using the Chi square test. HWE and genotype frequencies were calculated using Haploview software version 4.2 (Broad Institute, Cambridge, MA; available at http://www.broadinstitute.org/scientific-community/science/programs/medical-and-population-genetics/haploview/haploview).

Cumulative incidences of MCR, CCR, MMR, and MR4.5 were calculated consideration discontinuation of imatinib as a competing risk. Probabilities of freedom from LOR and TF were estimated and plotted using the Kaplan–Meier method. Probabilities of OS and PFS were also estimated using the Kaplan–Meier method. In univariate analyses, treatment outcomes, such as MCR, CCR, MMR, MR4.5, LOR, TF, PFS, and OS were compared using log-rank tests. Multivariate analysis was performed with variables including age, prior treatment, additional cytogenetic abnormalities, disease stage (CP versus AP/BC) and significant genotypes in the univariate analyses. The multivariate analyses using Cox’s proportional hazard models were conducted using an enter model and a *p* value for the likelihood ratio test of >0.05.

For validation of the genetic effect, we performed internal validation using a bootstrap algorithm. Bootstrap is a resampling technique which creates bootstrap data sets by sampling with replacement. It gave us a nonparametric maximal likelihood estimate of the prediction error and can correct for the bias of the estimate and therefore avoid the need for cross-validation. We applied bootstrap based on 1000 replications, and the results are presented as the bootstrap hazard ratio (HR), confidence intervals and *p* values of the genetic effects. All statistical tests were two-sided with the significance level set as 0.05.

The prognostic stratification value was assessed by the likelihood ratio test (LRT) using the Cox proportional hazard regression model for MR4.5 as the endpoint to measure how well the risk score performs in predicting future clinical events. The log likelihood ratio test evaluates superiority of a prognostic stratification system over another. Two multivariate models were compared: one with only clinical risk factors, and another using clinical risk factors as well as genotype data. All the above statistical tests were performed using SAS version 9.1 (SAS Institute, Cary NC, USA) and EZR (http://www.jichi.ac.jp/saitama-sct/SaitamaHP.files/statmedEN.html).

## Results

### Patient and disease characteristics and imatinib treatment outcomes

Patient demographic and disease characteristics are presented in Table 2. In total, 187 CML patients were included in the study. The median age was 49 years (range 11–87). One hundred and sixty nine patients (90 %) were in CP, 11 (6 %) in AP, and 7 (4 %) in BC. The median follow-up time was 41.7 months (range 1–98.1). Incidences of MCR at 6 months, CCR at 12 months, MMR at 18 months and MR4.5 at 3 years were 43.9, 63.1, 45.3 and 44.0 % respectively. Of the 187 patients, 53 had evidence of treatment failure, and 16 LOR.

**Table 2:** Summary of patient demographic and disease characteristics as well as treatment outcomes in 187 CML patients treated with imatinib

Characteristics	Variables	No of pts (%)
Gender	Female	79 (42)
	Male	109 (58)
Age	(Years, median, range)	49.0 (11–87)
Follow-up duration	(Months, median, range)	41.7 (0–98.1)
Cytogenetics at diagnosis	t(9;22) only	153 (81)
	Additional abnormalities^*^	27 (15)
	Unknown	7 (4)
Sokal risk group at diagnosis	Low	59 (32)
	Intermediate	69 (37)
	High	42 (22)
	Unknown	17 (9)
Disease stage at imatinib therapy	CP	169 (90)
	AP	11 (6)
	BC	7 (4)
Previous treatment (any)	De novo	145 (77.5)
	Previously treated	42 (22.5)
Previous treatment prior to imatinib	Interferon-alpha	30 (16.0)
Duration of imatinib therapy	Median in months (range)	36.9 (0–98.1)

*AP* accelerated phase,* BC* blastic crisis,* CP* chronic phase

* Additional cytogenetic abnormalities: t(9; 22; 11) (n = 1); t(7; 9; 22) (n = 1); t(9; 22; 17) (n = 1); t(9; 22; 19) (n = 1); t(4; 22) with t(17; 20) (n = 1); inv(3) (n = 2); der(9) (n = 1); der(9), del(9) (n = 1); del(22q) (n = 1); +der(22) (n = 3); −Y(n = 1); +8(n = 1); −12 (n = 1); 92, idemx2(n = 3); 69, XXX with 92, XXXX and 138, XXXXXX (n = 1), t(8;9;22) (n = 1); dup(1) (n = 1)

### Univariate analysis of candidate SNPs in the apoptosis pathway

Details of the eight candidate SNPs are summarized in Table 1 and Additional file 1: Table S1. There is a strong linkage disequilibrium between the SNPs in *FAS* (r2 = 1; rs2234767 and rs1800682), presented in Additional file 1: Figure S1.

The patient clinical characteristics, treatment history prior to imatinib, initial imatinib dose, disease stage and sokal risk score all correlated with responses to imatinib. In univariate analysis, patients who had prior treatment showed a lower probability of achieving MCR (p = 0.009), CCR (p = 0.016), MMR (p = 0.002), and MR4.5 (p = 0.011), and had a shorter time to TF (p = 0.039). Patients with a high sokal risk score showed a lower probability of achieving CCR (p = 0.050) and a shorter time to TF (p = 0.003) (Additional file 1: Table S2).

**Fig. 1:**
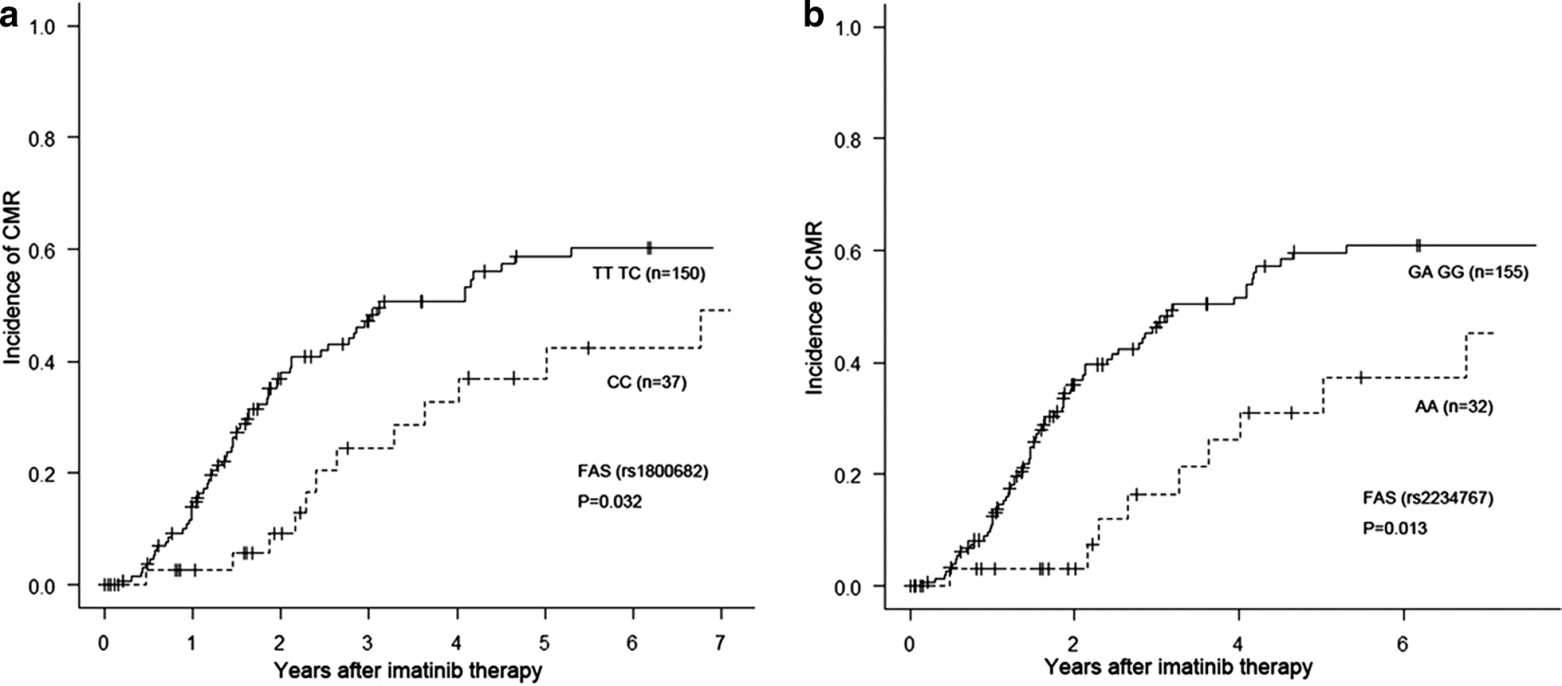
Incidence of MR4.5 based on the FAS (rs1800682) (**a**) and FAS (rs2234767) (**b**) genotypes

The results of univariate analyses for treatment outcomes with imatinib are presented in Table 3. The GG/GA versus AA *FAS* genotypes (rs2234767) showed rates of MR4.5 at 5 years of 59.7 ± 4.7 % (49.7–68.3 %) vs 37.4 ± 10.6 % (16.6–58.3 %) respectively (*p* value = 0.013, Hazard ratio 0.423, 95 % confidence interval [95 % CI 0.229–0.781]; Fig. 1b). The CC *FAS* genotype (rs1800682) also showed a lower MR4.5 rate than other genotypes (p = 0.032; Table 3 and Fig. 1). The incidence of MR4.5 at 5 years in the TT/TC *FAS* genotype (rs1800682) was 58.8 ± 5.1 % (48.2–68.0 %) vs 42.2 ± 10.0 % (22.1–61.3 %) in the CC genotype (HR 0.508, 95 % CI [0.291–0.886]; Fig. 1a).

**Table 3:** Summary of results of the univariate analysis for treatment outcomes based on the candidate genotypes in the apoptosis pathway

Gene	SNP ID	Referent genotype	Adverse genotype	MCR	CCR	MMR	MR4.5	LOR	TF	PFS	OS
*APAF1*	rs1439123	CC/CT	TT	0.803	0.393	0.233	0.085	0.011^*^	0.323	0.510	0.939
*APAF1*	rs2288713	GG/TG	TT	0.747	0.886	0.617	0.530	0.812	0.992	0.271	0.863
*CASP10*	rs13006529	AA/TA	TT	0.250	0.433	0.513	0.671	0.144	0.025^*^	0.311	0.019^*^
*FASLG*	rs763110	TT/TC	CC	0.414	0.186	0.017^*^	0.123	0.145	0.097	0.001^**^	0.020^*^
*FAS*	rs1800682	TT/TC	CC	0.871	0.354	0.251	0.032^*^	0.086	0.129	0.428	0.863
*FAS*	rs2229521	GG/GA	AA	0.856	0.688	0.748	0.863	0.600	0.384	0.600	0.685
*FAS*	rs2234767	GG/GA	AA	0.944	0.259	0.179	0.013^*^	0.034^*^	0.093	0.374	0.978
*FAS*	rs2234978	TT/TC	CC	0.026^*^	0.036^*^	0.050^*^	0.011^*^	0.379	0.089	0.409	0.535

*SNP* single nucleotide polymorphism, *MCR* major cytogenetic response, *CCR* complete cytogenetic response, *MMR* major molecular response, *MR4.5* complete molecular response, *LOR* loss of response, *TF* treatment failure, *PFS* progression free survival, *OS* overall survival, *APAF1* apoptotic peptidase activating factor 1, *CASP10* caspase 10, *FAS* Fas cell surface death receptor, *FASLG* Fas ligand (TNF receptor superfamily member 6)

** p < 0.01/* p < 0.05

**Fig. 2:**
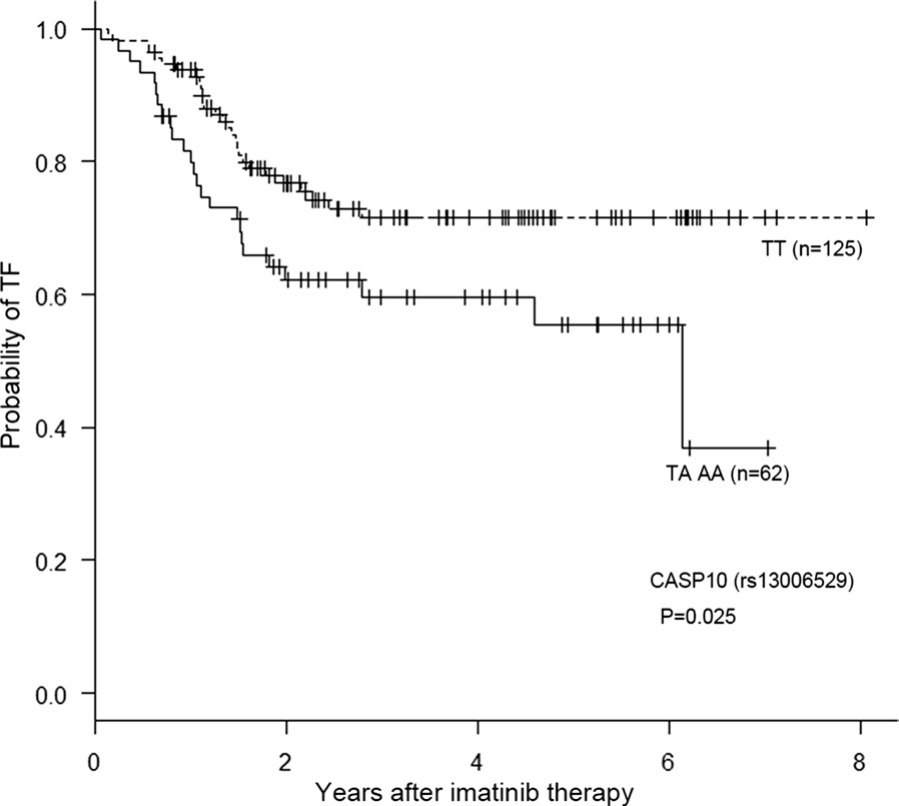
Probability of freedom from treatment failure based on the CASP10 genotype (13006529)

In the case of *CASP10* (rs13006529), the TT genotype correlated with a lower risk of TF and a longer OS than the TA/AA genotype (Table 3). The probability of freedom from TF at 5 years in the TT genotype, was 71.5 ± 4.7 % (61.2–79.5 %), vs 59.6 ± 6.6 % (45.2–71.2 %) in the TA or AA genotypes (p = 0.025, HR 0.543, 95 % CI [0.317–0.932]; Fig. 2).

**Fig. 3:**
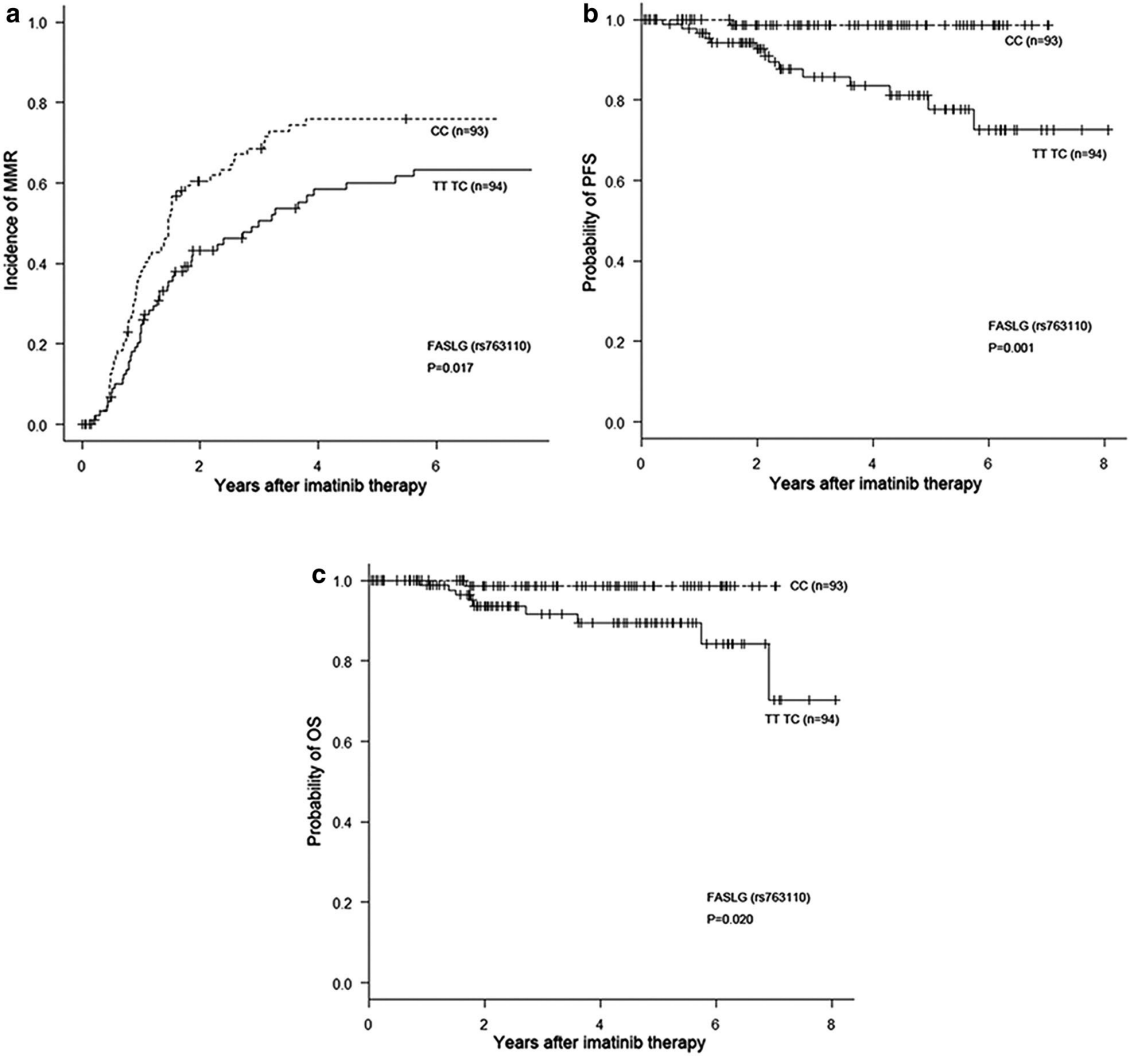
Differences in MMR (**a**), PFS (**b**) and OS (**c**) based on the FASLG genotype (rs763110)

Patients with the CC *FASLG* genotype (rs763110) showed a higher MMR rate, and a longer PFS and OS compared to the TT/TC genotype (Table 3 and Fig. 3): MMR at 18 months of 51.0 ± 5.4 % (39.9–61.0 %) vs 35.6 ± 5.1 % (25.6–45.7 %), p = 0.017; PFS of 98.7 ± 2.2 % (91.0–99.8 %) vs 77.7 ± 6.0 % (63.3–87.0 %), p = 0.001; and OS at 5 years of 98.6 ± 2.4 % (90.5–99.8 %) vs 89.5 ± 4.2 % (78.7–95.0 %) p = 0.020.

In *FAS* (rs2234978), the TT/TC genotype showed a higher rate of MCR (p = 0.026), CCR (p = 0.036), MMR (p = 0.050) and MR4.5 (p = 0.011) compared to those with the CC genotype (Table 3).

### Internal validation using a bootstrap procedure confirmed the significance of the correlation between *FAS* genotype and MR4.5

The p value from the bootstrap procedure was significant at 0.050 (95 % CI [0.000–0.221]), confirming that the *FAS*GG/GA genotype (rs2234767) was associated with a higher MR4.5 rate compared to the AA genotype (HR 0.431, 95 % CI [0.219–0.682]; Table 4).

**Table 4:** Internal validation using a Bootstrap procedure with 1000 replications

Parameter	Gene	SNP ID	Bootstrap *p* value (95 % CI)
MCR	FAS	rs2234978	0.138 (0.000–0.605)
CCR	FAS	rs2234978	0.187 (0.000–0.727)
MMR	*FASLG*	rs763110	0.065 (0.000–0.351)
	FAS	rs2234978	0.178 (0.000–0.762)
MR4.5	*FAS*	rs1800682	0.497 (0.053–0.951)
	FAS	rs2234767	0.050 (0.000–0.221)^*^
	FAS	rs2234978	0.073 (0.000–0.345)
LOR	*APAF1*	rs1439123	0.142 (0.000–0.749)
	FAS	rs2234767	0.169 (0.000–0.783)
TF	CASP10	rs13006529	0.102 (0.000–0.563)
PFS	*FASLG*	rs763110	0.365 (0.003–0.992)
OS	CASP10	rs13006529	0.162 (0.002–0.991)
	*FASLG*	rs763110	0.424 (0.017–0.994)

*MCR* major cytogenetic response, *CCR* complete cytogenetic response, *MMR* major molecular response, *MR4.5* complete molecular response, *LOR* loss of response, *TF* treatment failure, *PFS* progression free survival, *OS* overall survival, *HNF4A* hepatocyte nuclear factor 4, alpha, *APAF1* apoptotic peptidase activating factor 1, *FAS* Fas cell surface death receptor, *FASLG* Fas ligand (TNF receptor superfamily member 6)

* P ≤ 0.05

### Multivariate analyses confirming *FAS* genotype (rs2234767) as an independent predictor for MR4.5

Multivariate analysis was performed in order to confirm that the candidate SNP is an independent risk factor for clinical outcomes after adjustment for other clinical risk factors including age, prior treatment, additional cytogenetic abnormalities and disease stage.

Analysis revealed that the patients with the *FAS* AA genotype (rs2234767) showed a lower probability of achieving MR4.5 (p = 0.019, HR 0.43, 95 % CI [0.22–0.87]) than those with the GG/GA genotype, and that those with the CC genotype (rs2234978) had a lower probability of MR4.5 (p = 0.003 HR 0.45, 95 % CI [0.26–0.77]) than those with the CC genotype (Table 5).

**Table 5:** Results of multivariate analyses

Risk factor	Referent parameter	Adverse parameter	Univariate p value	Multivariate p value	HR (95 % CI)
MCR					
FAS (rs2234978)	CT/TT	CC	0.026	0.062	0.52 (0.26–1.03)
Age, continuous	–	–	0.748	0.920	1.00 (0.99–1.01)
Prior treatment	No	Yes	0.009	0.060	0.56 (0.30–1.03)
ACA	Absent	Present	0.128	0.560	0.85 (0.50–1.45)
Disease stage	AP/BC	CP	0.450	0.190	1.98 (0.72–5.46)
CCR					
FAS (rs2234978)	CT/TT	CC	0.036	0.100	0.56 (0.28–1.12)
Age, continuous	–	–	0.796	0.910	0.99 (0.98–1.01)
Prior treatment	No	Yes	0.016	0.074	0.57 (0.31–1.05)
ACA	Absent	Present	0.496	0.330	0.77 (0.45–1.30)
Disease stage	AP/BC	CP	0.399	0.060	2.48 (0.96–6.38)
MMR					
FAS (rs2234978)	CT/TT	CC	0.050	0.022^*^	0.53 (0.30–0.91)
*FASLG* (rs763110)	CT/TT	CC	0.017	0.079	1.42 (0.96–2.11)
Age, continuous	–	–	0.670	0.880	0.99 (0.98–1.01)
Prior treatment	No	Yes	0.002	0.007^**^	0.44 (0.24–0.80)
ACA	Absent	Present	0.097	0.490	0.81 (0.44–1.48)
Disease stage	AP/BC	CP	0.235	0.062	2.48 (0.96–6.44)
MR4.5					
*FAS* (rs2234767)	GA/GG	AA	0.013	0.019^*^	0.43 (0.22–0.87)
*FAS* (rs2234978)	CT/TT	CC	0.011	0.003^**^	0.45 (0.26–0.77)
Age, continuous	–	–	0.911	0.470	0.99 (0.98–1.01)
Prior treatment	No	Yes	0.011	0.051	0.50 (0.25–1.00)
ACA	Absent	Present	0.110	0.930	0.97 (0.46–2.02)
Disease stage	AP/BC	CP	0.316	0.110	3.17 (0.76–13.17)
LOR					
*APAF1* (rs1439123)	TT	CT/CC	0.011	0.174	0.36 (0.08–1.57)
FAS (rs2234767)	GA/GG	AA	0.034	0.177	2.21 (0.70–7.02)
Age, continuous	–	–		0.875	0.99 (0.96–1.03)
Prior treatment	No	Yes	0.062	0.538	1.47 (0.43–5.06)
ACA	Absent	Present	0.973	0.918	1.08 (0.24–4.89)
Disease stage	AP/BC	CP	0.432	0.607	1.77 (0.20–15.43)
TF					
CASP10 (rs13006529)	TA/AA	TT	0.025	0.049^*^	0.57 (0.32–0.99)
Age, continuous	–	–	0.978	0.869	0.99 (0.97–1.02)
Prior treatment	No	Yes	0.039	0.040^*^	1.94 (1.03–3.67)
ACA	Absent	Present	0.257	0.483	1.31 (0.61–2.81)
Disease stage	AP/BC	CP	0.001	0.093	0.47 (0.20–1.13)
PFS					
*FASLG* (rs763110)	CT/TT	CC	0.001	0.003^**^	0.04 (0.01–0.36)
Age, continuous	–	–	0.765	0.260	0.98 (0.94–1.02)
Prior treatment	No	Yes	0.235	0.053	3.28 (0.98–10.90)
ACA	Absent	Present	0.093	0.018^*^	4.36 (1.29–14.71)
Disease stage	AP/BC	CP	0.873	0.251	3.58 (0.41–31.68)
OS					
CASP10 (rs13006529)	TA/AA	TT	0.019	0.050^*^	0.25 (0.06–1.00)
*FASLG* (rs763110)	CT/TT	CC	0.020	0.046^*^	0.12 (0.01–0.97)
Age, continuous	–	–	0.787	0.300	0.97 (0.92–1.02)
Prior treatment	No	Yes	0.225	0.170	2.75 (0.65–11.64)
ACA	Absent	Present	0.441	0.175	3.07 (0.61–15.51)
Disease stage	AP/BC	CP	0.917	0.377	2.94 (0.27–32.15)

*ACA* additional cytogenetic abnormality, *HR* hazard ratio, *SNP* single nucleotide polymorphism, *MCR* major cytogenetic response, *CCR* complete cytogenetic response, *MMR* major molecular response, *MR4.5* molecular response with 4.5 log reduction, *LOR* loss of response, *TF* treatment failure, *PFS* progression free survival, *OS* overall survival, *APAF1* apoptotic peptidase activating factor 1, *CASP10* caspase 10, *FAS* Fas cell surface death receptor, *FASLG* Fas ligand (TNF receptor superfamily member 6)

* P < 0.05/** P < 0.01

Patients with the *CASP10* TT genotype (rs13006529) had a lower probability of freedom from TF (p = 0.049 HR 0.57, 95 % CI [0.32–0.99]) and a better OS (p = 0.050 HR 0.25, 95 % CI [0.06–1.00]) than those with the AA/TA genotype. In addition, patients with the *FASLG* CC genotype (rs763110) had a better PFS (p = 0.003 HR 0.04, 95 % CI [0.01–0.36]) and OS (p = 0.046 HR 0.12, 95 % CI [0.01–0.97]) than those with the TT/TC genotype. History of treatment prior to imatinib was a risk factor for TF (p = 0.040 HR 1.94, 95 % CI [1.03–3.67]) and MMR (p = 0.007 HR 0.44, 95 % CI [0.24–0.80]; Table 5).

### The incorporation of genotype in addition to clinical factors in multivariate analysis improved the prognostic stratification power for MR4.5

We attempted to answer the question of whether the addition of genotype data could enhance prognostic stratification. Two multivariate models were compared: one with clinical risk factors alone, and another with additional genotype data. The log likelihood ratio test for MR4.5 showed a significant difference between the two models in favor of the model including genotype data (p = 0.006; Additional file 1: Table S3).

## Discussion

Imatinib is a specific inhibitor of the bcr-abl1 fusion protein and is an example of successful targeted therapy [29]. However, it appears that the clinical response of imatinib relies on the genetic background of a patient. In this study we focused on eight SNPs involved in the apoptosis pathway. Results showed that: (1) the GG/GA *FAS* genotype (rs2234767) showed a higher MR4.5 rate at 5 years of 59.7 % compared to the AA genotype (37.4 %, p = 0.013); (2) an internal validation procedure confirmed that *FAS* (rs2234767) correlates with MR4.5 using multiple replications with a bootstrap algorithm (p = 0.050); (3) multivariate analyses confirmed that *FAS* genotype (rs2234767) is an independent predictor for MR4.5 even after taking into account other clinical risk factors including age, prior treatment, additional cytogenetic abnormality and disease stage (p = 0.019, HR 0.43, 95 % CI [0.22–0.87]). Recent advances in the CML treatment suggested around 40 % of patients can achieve treatment free remission without requiring active CML treatment even after stopping TKI in the patients attained deeper molecular response (defined as 4.5 log reduction of bcr-abl1 transcript or deeper) for 2 years or longer following imatinib therapy for 5 years or longer. This finding emphasized clinical importance of MR4.5 achievement as a relevant milestone for TKI discontinuation attempt [30].

Binding of Fas ligand (FasL) to its membrane receptor Fas-R induces apoptosis via a typical death receptor signaling pathway. The trimerization of Fas-R upon the binding with FasL recruits and subsequently activates Caspase 8 and 10, and these initiator caspases trigger apoptosis by activating executive Caspase 3 directly, or by activating Bid and releasing cytochrome C from mitochondria. The FasL/Fas-R signaling pathway plays an important role in both physiological and pathological conditions [31]. Fas-R is expressed constitutively in CD34+ cells in CML patients. Fas-R triggering results in a decreased proliferation rate due to apoptosis of clonogenic cells [32].

The Fas-mediated apoptosis pathway is important in imatinib’s mechanism of action. The *FAS* genotype (rs2234767) showed a significant and independent association with the achievement of MR4.5. Furthermore the *FAS* genotype showed a greater correlation with MR4.5 than other clinical parameters, probably because the Fas-mediated apoptosis pathway is more significant in CD34+/CD38− LSCs compared to differentiated hematopoietic cells, a parameter reflecting LSC burden after imatinib therapy.

The two SNPs in *FAS*, rs2234767 (GG/GA vs AA) and (rs1800682; TT/TC vs CC) that had a strong association with MR4.5 are functional polymorphisms in the *FAS* promoter region. The A to G transition at rs1800682 in the enhancer region, modulates the signal transducer and activator of transcription (STAT-1), thus diminishing promoter activity and resulting in down-regulation of Fas expression [33]. The G to A substitution at rs2234767 in the silencer region of the *FAS* gene is reported to be associated with cancer risk, so that the AA genotype increases the risk of cancer, while the G-allele is a protective factor [34]. In this study, the G allele increased the rate of MR4.5 compared to the A allele. This suggests that the G-allele may result in higher Fas expression that correlates with a better response to imatinib therapy.

Other genotypes also showed potential correlations with clinical outcomes of imatinib therapy. The *CASP10* genotype (rs13006529) which encodes an immediate downstream apoptotic initiator CASP10 was associated with treatment failure. Although this SNP lacks functional validation it is known to be significantly associated with cancer risk [35]. Also, SNPs in *FASLG*, rs763110 (CC vs TC + TT) showed a strong association with MMR, PFS and OS. These SNPs are located within a putative binding motif in the CAAT/enhancer-binding protein β transcription factor. Higher expression of *FASLG* is associated with the C allele compared to the T allele, thus the polymorphism may affect FasL expression and therefore FasL/Fas signaling [36].

One limitation of this study is that we only analyzed the association between these SNPs and outcomes of imatinib in Korean patients. As the genetic backgrounds among different ethnic populations may differ significantly, other SNPs in these genes and others involved in the apoptosis pathways may also be relevant to the efficacy of imatinib treatment in those groups and would need to be investigated further. In conclusion, treatment outcomes of imatinib therapy in CML patients correlate well with genetic variants in the apoptosis pathway. The Fas/FasL signaling pathway may represent the major pathway that mediates apoptosis in CML treated with imatinib. The response to imatinib, particularly MR4.5, was strongly associated with the *FAS* genotype (rs2234767) which was confirmed in multivariate analyses and also validated using a Bootstrap procedure. These apoptosis-associated SNPs can be used as predictive/prognostic markers for imatinib treatment in CML patients allowing more risk adaptive strategies in patients at risk of treatment failure. Furthermore, additional strategies that enhance the Fas/FasL signaling pathway with imatinib may increase its clinical efficacy. The weakness of the present study is lack of replication data using an independent set of patients. Accordingly, validation of the current results will be needed in an independent set of patients with different ethnicities in order to reach clear conclusion on the issue. Further study is also necessary to identify the functional aspects of these SNPs in relation to the mechanism of action of imatinib, especially in LSCs and to identify variations in different ethnic groups.

## Conclusions

In summary, we analyzed the association between SNPs in the Fas/FasL signaling pathway and treatment outcomes including molecular response with 4.5 log reduction (MR4.5), following imatinib therapy in 187 Korean CML patients. We identified the Fas genotype (rs2234767) as a potential independent surrogate for MR4.5 in imatinib therapy. Genetic testing and some specific SNPs are helpful for drug selection for CML patients.

## Additional file

10.1186/s12967-016-0837-5 **Figure S1.** Linkage disequilibrium plot among the 8 genotypes in the apoptosis pathway: There is a strong linkage disequilibrium between the SNPs in FAS (r2 = 1; rs2234767 and rs1800682) . **Table S1.** The primer design used in genotyping of 8 candidate gene single nucleotide polymorphisms. **Table S2.** P-values of univariate analysis for imatinib treatment outcomes based on clinical variables. **Table S3.** Results of log likelihood ratio test comparing prognostic models including clinical factors alone and the addition of genotype data.

## Abbreviations

SNPs: single nucleotide polymorphisms
MR4.5: molecular response with 4.5 log reduction
CML: chronic myeloid leukemia
LSCs: leukemic stem cells
TKIs: tyrosine kinase inhibitors
HSCs: hematopoietic stem cells
MALDI-TOF: matrix-assisted laser desorption/ionization time-of-flight
CCR: complete cytogenetic response
MCR: major cytogenetic response
MMR: major molecular response
TF: treatment failure
LOR: loss of response
CP: chronic phase
AP: accelerated phase
BC: blastic crisis
PFS: progression free survival
OS: overall survival
HWE: Hardy–Weinberg Equilibrium
HR: hazard ratio
LRT: likelihood ratio test
