# Supplementary material for: Single nucleotide polymorphisms in apoptosis pathway are associated with response to imatinib therapy in chronic myeloid leukemia
Source: J Transl Med. 2016 Mar 24;14:82. doi: 10.1186/s12967-016-0837-5 (PMC4806489; doi:10.1186/s12967-016-0837-5)
Supplement: Supplementary file 1 — 10.1186/s12967-016-0837-5 Figure S1. Linkage disequilibrium plot among the 8 genotypes in the apoptosis pathway: There is a strong linkage disequilibrium between the SNPs in FAS (r2 = 1; rs2234767 and rs1800682) . Table S1. The primer design used in genotyping of 8 candidate gene single nucleotide polymorphisms. Table S2. P-values of univariate analysis for imatinib treatment outcomes based on clinical variables. Table S3. Results of log likelihood ratio test comparing prognostic models including clinical factors alone and the addition of genotype data. [file 12967_2016_837_MOESM1_ESM.doc]

Fig S1 Linkage disequilibrium plot among the 8 genotypes in the apoptosis pathway


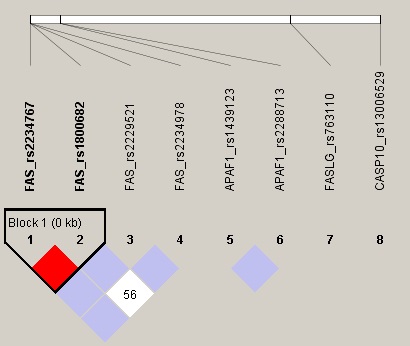


**Table S1 The primer design used in genotyping of 8 candidate gene single nucleotide polymorphisms**

| Gene | SNP ID | PCR primer 1 | PCR Primer 2 | Extension Primer |
| --- | --- | --- | --- | --- |
| *APAF1* | rs1439123 | ACGTTGGATGTAGTCTGAGAGAAACCCAGC | ACGTTGGATGAGGACGCAGATTCAGAGATG | AAGCGTACATTAAACTGGGAACAGTA |
| *APAF1* | rs2288713 | ACGTTGGATGGCTGATTCTAGCAAAGGAAC | ACGTTGGATGAATCTGAATGTATGCCCAAC | CTCTTAATGTATGCCCAACGTAATG |
| *CASP10* | rs13006529 | ACGTTGGATGGCTGGAGGTTATAGCCAATG | ACGTTGGATGAACTAGTATTCCCTGTGCCC | GCCCCTGGATGCACTTTCA |
| *FASLG* | rs763110 | ACGTTGGATGCTGGGCAAACAATGAAAATG | ACGTTGGATGTAGATCAGAGGCTGCAAACC | GGACACACAGAGCTGCTTTGTATTTC |
| *FAS* | rs1800682 | ACGTTGGATGTTGTGGCTGCAACATGAGAG | ACGTTGGATGTCCCTTTTCAGAGCCCTATG | CCGCATATGGTTAACTGTCCATTCCAG |
| *FAS* | rs2229521 | ACGTTGGATGTGTACTCCTTCCCTTCTTGG | ACGTTGGATGAGGTGAAAGGAAAGCTAGGG | GGGTAAAGCTAGGGACTGCAC |
| *FAS* | rs2234767 | ACGTTGGATGGTTAGTGCCATGAGGAAGAC | ACGTTGGATGCATCCTCCTTATCCCACTTC | GTGCACAAGGCTGGCAC |
| *FAS* | rs2234978 | ACGTTGGATGCACAGAAAGGAAAACCAAGG | ACGTTGGATGTTTCAAGGAAAGCTGATACC | TATTTCAATACCTACAGGATTTAA |

Abbreviations: SNP, single nucleotide polymorphism; PCR, polymerase chain reaction; MCR, major cytogenetic response; CCR, complete cytogenetic response; MMR, major molecular response; CMR, complete molecular response; LOR, loss of response; TF, treatment failure; PFS, progression free survival; OS, overall survival; *APAF1*, apoptotic peptidase activating factor 1; *CASP10*, caspase 10; *FAS*, Fas cell surface death receptor; *FASLG*, Fas ligand (TNF receptor superfamily member 6).

**Table S2 P-values of univariate analysis for imatinib treatment outcomes based on clinical variables**

|  | Referent parameter (n) | Adverse parameter (n) | MCR | CCR | MMR | MR4.5 | LOR | TF | PFS | OS |
| --- | --- | --- | --- | --- | --- | --- | --- | --- | --- | --- |
| Gender | Male (109) | Female (79) | 0.105 | 0.059 | 0.073 | 0.050 | 0.762 | 0.566 | 0.989 | 0.972 |
| Prior treatment | Absence (187) | Presence (30) | 0.009^**^ | 0.016^*^ | 0.002^**^ | 0.011^*^ | 0.062 | 0.039^*^ | 0.235 | 0.225 |
| ACA | Absence (160) | Presence (27) | 0.128 | 0.496 | 0.097 | 0.110 | 0.973 | 0.257 | 0.093 | 0.441 |
| Sokal score | Low/intermediate(128) | High (42) | 0.728 | 0.050^*^ | 0.113 | 0.195 | 0.288 | 0.003^**^ | 0.267 | 0.107 |
| Disease stage | CP(167) | AP/BC (18) | 0.450 | 0.399 | 0.235 | 0.316 | 0.432 | 0.001^**^ | 0.873 | 0.917 |

Abbreviations: MCR, major cytogenetic response; CCR, complete cytogenetic response; MMR, major molecular response; MR4.5, complete molecular response; LOR, loss of response; TF, treatment failure; PFS, progression free survival; OS, overall survival; Allo-HSCT, allogeneic hematopoietic stem cell transplantation; IM, imatinib; ACA, additional cytogenetic abnormality; AP, accelerated phase; BC, blastic crisis; CP, chronic phase.

**p<0.01/*p<0.05

**Table S3 Results of log likelihood ratio test comparing prognostic models including clinical factors alone and the addition of genotype data**

|  | p-value | Likelihood ratio in model incorporating clinical factors and genotype data | Likelihood ratio in model using clinical factors only | *Χ^2^*of likelihood ratio test comparing two models |
| --- | --- | --- | --- | --- |
| MCR | 0.104 | 11.37 | 8.72 | 2.65 |
| CCR | 0.144 | 12.91 | 10.78 | 2.13 |
| MMR | 0.051 | 20.56 | 14.61 | 5.95 |
| MR4.5 | 0.006^*^ | 22.76 | 12.28 | 10.48 |
| LOR | 0.068 | 7.04 | 1.66 | 5.37 |
| TF | 0.177 | 8.55 | 6.73 | 1.82 |
| PFS | <0.001^*^ | 17.46 | 2.51 | 14.94 |
| OS | 0.003^*^ | 12.83 | 1.40 | 11.43 |

Abbreviations: MCR, major cytogenetic response; CCR, complete cytogenetic response; MMR, major molecular response; MR4.5, complete molecular response; LOR, loss of response; TF, treatment failure; PFS, progression free survival; OS, overall survival.

*p<0.01
